# Comparison of biofilm formation and motility processes in arsenic‐resistant *Thiomonas* spp. strains revealed divergent response to arsenite

**DOI:** 10.1111/1751-7915.12556

**Published:** 2017-02-07

**Authors:** Julien Farasin, Sandrine Koechler, Hugo Varet, Julien Deschamps, Marie‐Agnès Dillies, Caroline Proux, Mathieu Erhardt, Aline Huber, Bernd Jagla, Romain Briandet, Jean‐Yves Coppée, Florence Arsène‐Ploetze

**Affiliations:** ^1^ Laboratoire Génétique moléculaireGénomique et MicrobiologieUMR7156CNRS and Université de StrasbourgInstitut de BotaniqueStrasbourgFrance; ^2^ Institut PasteurPlate‐forme Transcriptome et EpigenomeBioMicsCentre d'innovation et recherche technologiqueParisFrance; ^3^ Institut PasteurHub Bioinformatique et BiostatistiqueCentre de BioinformatiqueBiostatistique et Biologie Intégrative (C3BI, USR 3756, IP CNRS)ParisFrance; ^4^ Micalis InstituteINRAAgroParisTechUniversité Paris‐SaclayJouy‐en‐JosasFrance; ^5^ Université de StrasbourgCNRSIBMP UPR 2357F‐67000 StrasbourgFrance; ^6^ Present address: Géosciences Rennes – UMR 6118Université de Rennes 1RennesFrance; ^7^ Present address: Université de StrasbourgCNRSIBMP UPR 2357F‐67000StrasbourgFrance; ^8^ Present address: UPR3212Institut des neurosciences cellulaires et integrativesCNRSUniversité de StrasbourgStrasbourgFrance; ^9^ Present address: Center for Human ImmunologyCRT & Hub de Bioinformatique et BiostatistiquesC3BIInstitut PasteurParis France

## Abstract

Bacteria of the genus *Thiomonas* are found ubiquitously in arsenic contaminated waters such as acid mine drainage (AMD), where they contribute to the precipitation and the natural bioremediation of arsenic. In these environments, these bacteria have developed a large range of resistance strategies among which the capacity to form particular biofilm structures. The biofilm formation is one of the most ubiquitous adaptive response observed in prokaryotes to various stresses, such as those induced in the presence of toxic compounds. This study focused on the process of biofilm formation in three *Thiomonas* strains (CB1, CB2 and CB3) isolated from the same AMD. The results obtained here show that these bacteria are all capable of forming biofilms, but the architecture and the kinetics of formation of these biofilms differ depending on whether arsenite is present in the environment and from one strain to another. Indeed, two strains favoured biofilm formation, whereas one favoured motility in the presence of arsenite. To identify the underlying mechanisms, the patterns of expression of some genes possibly involved in the process of biofilm formation were investigated in *Thiomonas* sp. CB2 in the presence and absence of arsenite, using a transcriptomic approach (RNA‐seq). The findings obtained here shed interesting light on how the formation of biofilms, and the motility processes contribute to the adaptation of *Thiomonas* strains to extreme environments.

## Introduction

Despite its low crustal abundance, arsenic occurs ubiquitously around the world, often in association with various minerals (Mandal and Suzuki, 2002; Lièvremont *et al*., 2009; Oremland *et al*., 2009; Sharma and Sohn, 2009). As arsenic is highly soluble in water with a pH value close to neutral, water supplies are frequently contaminated, and this toxic element is known to be a major risk to human health in many parts of the world (Lièvremont *et al*., 2009). Arsenic disturbs the metabolism of most organisms and is thought to be mutagenic, carcinogenic and teratogenic. The highly toxic inorganic forms of arsenic often predominate in aqueous environments (Mandal and Suzuki, 2002; Oremland and Stolz, 2005; Lièvremont *et al*., 2009) such as geothermal sources and water mine drainage (acid mine drainage or AMD) (Mandal and Suzuki, 2002; Oremland and Stolz, 2005), which also contain many other heavy metals and show generally highly acidic pH levels, which are usually below 3.

Many bacteria are able to withstand these extreme environments and colonize AMD because they have developed mechanisms for adapting to extreme conditions (Oremland and Stolz, 2005; Silver and Phung, 2005; Kruger *et al*., 2013). In particular, the arsenic resistance mechanisms include the expression of enzymes that either transform the toxic metal (as in the case of the arsenite oxidase AioBA), extrude it (as in the case of ArsB) or prevent its uptake by repressing the expression of low specificity phosphate transporter and inducing that of high specificity phosphate transporter (Pst) (Tsai *et al*., 2009; Bertin *et al*., 2012; Kruger *et al*., 2013). Previous results have suggested that arsenic‐resistant bacteria of various origins are able to form biofilms in the presence of arsenic (Michel *et al*., 2007, 2011; Denef *et al*., 2010; Marchal *et al*., 2010, 2011; Andres *et al*., 2013). In prokaryotes, biofilm formation is one of the main adaptive responses to various stresses. These highly structured communities of microorganisms enable cells to resist many environmental stresses such as extreme pH and oxygen levels, desiccation, and the presence of bactericidal compounds (Hall‐Stoodley *et al*., 2004). There are several reasons why microorganisms survive toxic compounds in biofilm better than in a planktonic state. The matrix of the biofilm, which is composed mainly of polysaccharides and proteins, isolates the cells from environmental stresses by limiting the spread of antibacterial compounds or by sequestering them. On the other hand, the high cell density of these communities promotes horizontal gene transfer processes and the acquisition of new features (Aminov, 2011). These events promote the emergence of cell variants that are more persistent or have greater survival skills than those of the starting population. Therefore, biofilm formation constitutes a collective strategy for surviving stress. Other adaptive responses such as swimming, twitching, swarming, gliding and floating motility constitute also strategies for surviving stress, because they enable cells to migrate actively to more suitable environments (Jarrell and McBride, 2008). Floating (moving vertically up a water column, propelled by gas vesicles) and gliding (described in *Mycoplasma*) do not seem to require any special appendages. On the contrary, swimming (individual cells moving in liquid) and swarming (multicellular movement of bacteria across a surface) are forms of motility which involve flagella, and twitching motility is performed on solid surfaces via Type IV pili (Jarrell and McBride, 2008). Soft agar motility tests (0.3% agar) are commonly used for quantifying swimming motility and testing chemotaxis: at this low agar concentrations, the bacteria can swim towards a more favourable zone (e.g. containing larger supplies of nutrients) or escape a zone with adverse conditions (e.g. presence of a toxic element). With higher agar concentrations (0.5–0.7%), the bacteria swarm over the agar surface (Kearns, 2010).

Biofilm formation and motility are two closely related processes and are therefore often analysed together. Once the cells have detected any toxic compounds in their environment, they can either escape by initiating a motility process, which is modulated via chemotaxis, or adhere to a surface and form biofilms (Harrison *et al*., 2007; Nagar and Schwarz, 2015). Interestingly, the flagella or Type IV pili involved in swimming, swarming or twitching motility are often also involved in the early stages of biofilm development (Stoodley *et al*., 2002; Hall‐Stoodley *et al*., 2004). Once the biofilm is formed, mobile subpopulations often emerge in the biofilm and disperse. It was recently established that mobile cells can also invade mature biofilms, creating transient pores that increase the nutrient flow in the matrix (Houry *et al*., 2012). These two processes are tightly controlled via complex transcriptional, translational and post‐translational regulatory mechanisms (Jarrell and McBride, 2008; Guttenplan and Kearns, 2013; Martínez and Vadyvaloo, 2014). Some regulatory pathways which are common to the two processes enable bacteria to switch from a sessile lifestyle in a biofilm to the mobile planktonic lifestyle, via quorum sensing and the second messenger molecule, cyclic‐di‐GMP, in particular (Martínez and Vadyvaloo, 2014; Hobley *et al*., 2015; Nagar and Schwarz, 2015).

We recently studied the genetic and functional diversities of several strains belonging to the *Thiomonas* genus (Moreira and Amils, 1997), which occurs ubiquitously in AMD and contributes to the transformation of metals and metalloids such as arsenic (Bryan *et al*., 2009; Arsène‐Ploetze *et al*., 2010; Marchal *et al*., 2011; Slyemi *et al*., 2011), and we compared their physiological characteristics (Bryan *et al*., 2009; Arsène‐Ploetze *et al*., 2010). In particular, genomic and physiological comparisons between several closely related *Thiomonas* strains isolated from the Carnoulès AMD showed that they differ in their resistance capacities and in terms of the presence of genomic islands (GEIs; Farasin *et al*., 2015; Freel *et al*., 2015). In one of these strains, *Tm*. sp. CB2, the process of biofilm formation was studied in the presence and absence of arsenite (As(III)) (Marchal *et al*., 2011). A particularly complex biofilm structure was observed in the presence of As(III). Other *Thiomonas* strains were isolated that may differ in their ability to swim or form biofilm (Bryan *et al*., 2009; Arsène‐Ploetze *et al*., 2010). In this study, we compared the ability of three *Thiomonas* strains (CB1, CB2 and CB3) to form biofilms and to develop motility responses, because these two processes are probably linked, and we studied the effects of As(III) on these processes. It was then proposed to search for any difference in genomic content due to the presence of GEIs, which might explain the differences between the responses observed. Lastly, we analysed the patterns of gene expression observed during the process of biofilms development induced by the presence of As(III) in the strain *Thiomonas* sp. CB2, using a RNA sequencing approach. Based on the results of these analyses, we discussed how biofilm formation can contributes in *Thiomonas* resistance to arsenic.

## Results

### Comparison between the biofilm development and motility responses occurring in *Thiomonas* strains

In the first set of analyses, the strains *Thiomonas* spp. CB1, CB2 and CB3, isolated from the same AMD, were tested by performing soft agar motility tests to determine whether the presence of arsenite (As(III)) affected their swimming motility or chemotaxis. For this purpose, diluted cell culture was deposited on soft agar supplemented or not with 2.67 or 5.33 mM of As(III). *Tm*. spp. CB1 strain was mobile only in the presence of As(III), showing that motility was induced in this strain in the presence of this element (Fig. S1A). No motility was observed in the case of *Tm*. spp. CB2 and CB3, whether or not As(III) was present. Strains *Tm*. spp. CB1 and CB2 were able to synthesize a flagellum in both the presence and absence of As(III) in the liquid growth medium (Fig. S1B). In contrast, none or a very short flagellum was observed in strain CB3, whether or not As(III) was present in the liquid growth medium (Fig. S1B).

A crystal violet staining method was carried out on biofilms grown for 72 h in 12‐well plates, in order to determine the ability of each of the strains to produce biofilms in response to the presence of As(III) (Fig. S2). The biofilms were cultivated in m126 supplemented or not with 2.67 or 5.33 mM of As(III), and the medium was renewed after 24 h of growth. Cells firmly attached on the plates and their biofilm matrix were stained with crystal violet and quantified by measuring the O.D at 595 nm. *Tm*. sp. CB1 did not adhere efficiently and/or produced relatively low amounts of biofilm. Moreover, no effect or a slight effect of As(III) on biofilm formation was observed for this strain. In contrast, *Tm*. spp. CB2 and CB3 were able to adhere or form biofilm more efficiently than *Tm*. sp. CB1 both in the absence and in the presence of 5.33 mM of As(III) (Fig. S2). We also observed that the production of biofilm matrix or the cell adhesion process may be regulated differently in these two strains in the presence of As(III), since at 5.33 mM As(III), as compared to 2.67 mM, the amount of attached cells and biofilm decreased in the case of *Tm*. sp. CB2, whereas it increased in the case of *Tm*. sp. CB3. Altogether, these first experiments revealed that As(III) induced the motility process but did not influence the biofilm formation in *Tm*. sp. CB1, whereas this toxic element seems to influence the biofilm formation but not the motility process in *Tm*. spp. CB2 and CB3.

To investigate more precisely the differences in biofilm development between *Tm*. spp. CB1, CB2 and CB3 as well as the effects of As(III) on this process, a confocal microscopy analysis of the biofilm architecture was performed after 24 and 72 h of growth in 12‐well plates. These time points were chosen because, as previously established with *Tm*. sp. CB2 (Marchal *et al*., 2011), the amount of attached cells could be quantified after 24 h, whereas the biofilms are mature after 72 h. The bacterial cells and extracellular polysaccharides were stained with SYTO9 and tetramethylrhodamine conjugate of concanavalin A (ConA), respectively, and their amount was quantified using ICY (http://icy.bioimageanalysis.org/) as described previously (Sanchez‐Vizuete *et al*., 2015).

**Figure 1:**
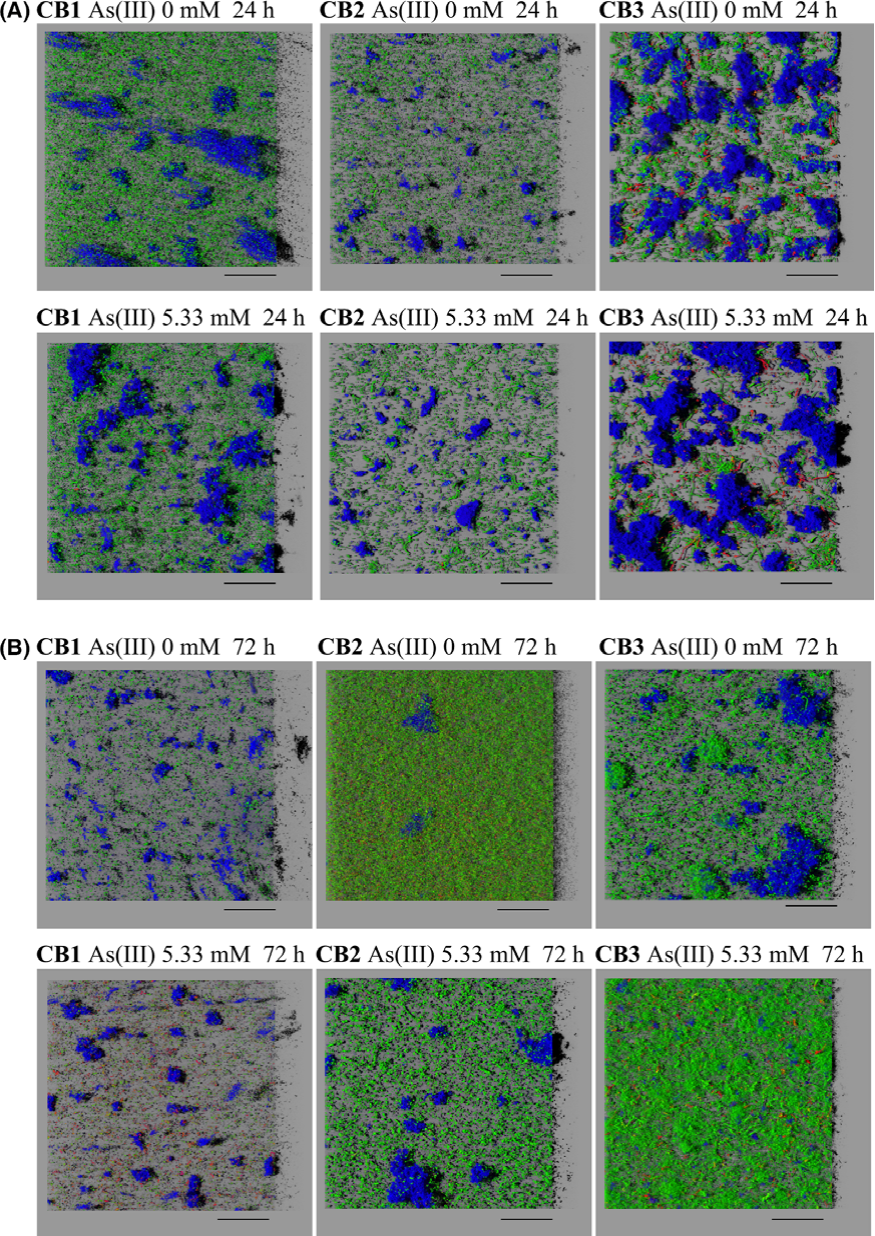
3D projections of biofilms obtained from confocal microscopy data (imaris software). Blue: exopolysaccharides matrix stained with concanavalin A conjugated to tetramethylrhodamine. Green: bacterial cells stained with SYTO9. Red: dead cells stained with propidium iodide. Scale bar: 3.10^4^ μm. A. 24 h. Microcolonies and many dense polysaccharide aggregates were observed, even after this short time of incubation. B. 72 h. The biovolume of cells was more important for *Tm*. sp. CB2, and these cells cover uniformly the surface. A high proportion of dead cells was observed in the biofilm made by *Tm*. sp. CB1, as compared to the other strains.

**Figure 2:**
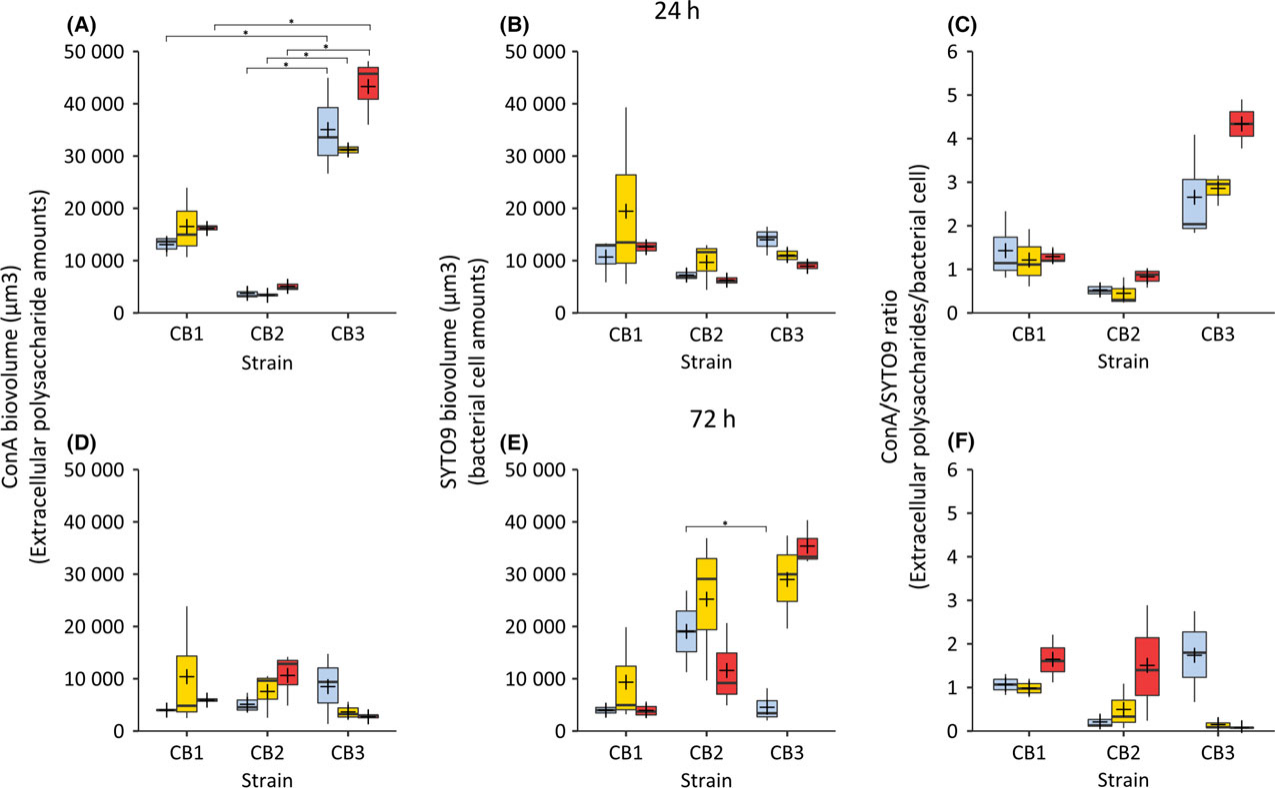
SYTO9 and tetramethylrhodamine conjugate of concanavalin A (ConA) biovolumes of biofilms. The extracellular polysaccharides and bacterial cells were labelled with ConA (tetramethylrhodamine conjugate of ConA; Life Technologies) and SYTO9 (SYTO ^®^ 9 green; Life Technologies), respectively, as previously described (Marchal *et al*., 2011). Blue: performed without As(III). Yellow and red: performed in the presence of 2.67 and 5.33 mM As(III) respectively. A–C. 24 h. *Tm*. sp. CB3 ConA biovolume is more important than *Tm*. spp. CB1 and CB2 (Tukey's test, *P* < 4 × 10^−4^, indicated by *). No difference in ConA or SYTO9 biovolumes was observed in the presence or in the absence of As(III), suggesting that As(III) has no effect on cell attachment and matrix biosynthesis at early stages of biofilm development. The ConA/SYTO9 ratio is higher for *Tm*. sp. CB3 than for the other two strains, suggesting a higher exopolysaccharides production capacity of this strain. D–F. 72 h. As compared to 24 h, the ConA biovolumes were lower for *Tm*. sp. CB3 (Tukey's test, *P* < 2 × 10^−5^) that could be due to a process of dispersal. In the absence of As(III), the SYTO9 biovolume was higher for *Tm*. sp. CB2 than for *Tm*. sp. CB3 (Tukey's test, *P* = 0.038, indicated by *) and for *Tm*. sp CB1. In the presence of As(III), the ConA/SYTO9 ratio increased in the case of *Tm*. spp. CB1 and CB2, but decreased in the case of *Tm*. sp. CB3.

The global analysis of the data revealed that the amount of bacterial cells (revealed by the SYTO9 biovolume) varied with the incubation time, depending on the strain and the presence or absence of As(III) (ANOVA, *P* = 0.021). The amount of extracellular polysaccharides (revealed by the ConA biovolume) depended on the incubation time and on the strain (ANOVA, *P* = 4.13 × 10^−12^). A detailed analysis of the biofilm development was then performed. After 24 h of growth, the cells formed aggregates resembling microcolonies with all three strains, and many dense polysaccharide aggregates were also observed (Fig. 1A). The amount of extracellular polysaccharides was larger in *Tm*. sp. CB3 than in *Tm*. spp. CB1 and CB2, both in the absence of As(III) (Fig. 2A; Tukey's test, *P* = 3.92 × 10^−4^ and *P* = 4.17 × 10^−7^ respectively) and in the presence of 5.33 mM As(III) (Fig. 2A; Tukey's test, *P* = 8.66 × 10^−6^ and *P* = 3.51 × 10^−9^ respectively). The amount of bacterial cells was similar in all three strains (Fig. 2B), and the amount of extracellular polysaccharides per bacterial cell (ConA/SYTO9 biovolume ratio) was therefore higher in *Tm*. sp. CB3 (Fig. 2C) than in the other two strains within 24 h of growth. At this time point, no significant As(III) effect was observed on either the amount of extracellular polysaccharides (Fig. 2A) or the amount of bacterial cells (Fig. 2B).

After 72 h of growth, many dense polysaccharide aggregates were observed in the three strains (Fig. 1B). In the absence of As(III), the *Tm*. sp. CB2 cells were spread uniformly over the surface (Fig. 1B) and their amount was greater than *Tm*. spp. CB1 and CB3 cell amounts (Fig. 2E; Tukey's test, *P* = 0.038). The amounts of extracellular polysaccharides were similar in the three species (Fig. 2D), and the amount of extracellular polysaccharides per bacterial cell was therefore higher in *Tm*. sp. CB3 than in *Tm*. sp. CB2 (Fig. 2F). In *Tm*. sp. CB2, the amount of extracellular polysaccharides was slightly higher in the presence than in the absence of As(III) (Fig. 2D). However, the amount of bacterial cells was not significantly affected by As(III) (Fig. 2E). Consequently, the amount of extracellular polysaccharides per bacterial cell was higher in the presence than in the absence of As(III) in the *Tm*. sp. CB2 strain (Fig. 2F). That was, however, not the case of *Tm*. sp. CB1, as, in this strain, As(III) had no significant effect on the amount of extracellular polysaccharides or on the amount of bacterial cell (Fig. 2D and E). A larger number of dead cells were observed in the biofilm formed by *Tm*. sp. CB1 than in the other strains (Fig. 1B). This strain therefore seems to be more sensitive to As(III) than the other two strains. In the case of *Tm*. sp. CB3, As(III) had a positive effect on the amount of attached cells but a negative effect on the amount of extracellular polysaccharides per bacterial cell. Surprisingly, the amount of extracellular polysaccharides at 72 h was lower than after 24 h in *Tm*. sp. CB3, independently of the As(III) (Fig. 2A and D; Tukey's test, *P* < 2 × 10^−5^). This decrease may have been due to the natural process of dispersal, as the dispersed cells may have been partly removed when the medium was renewed after 24 h.

In conclusion, the results obtained in this experiment show that the three *Thiomonas* strains respond differently to the presence of As(III), leading to the formation of different biofilm architectures. Indeed, no particularly marked effects of As(III) were observed in the case of *Tm*. sp. CB1, whereas in that of *Tm*. sp. CB2, As(III) seems to have negative effects on the cell growth and/or adhesion processes (Fig. 2E) but positive effects on the exopolysaccharide production rate (as observed after 72 h of growth, Fig. 2D and F). This increase in polysaccharide production in the presence of As(III) had already been demonstrated in *Tm*. sp. CB2 (Marchal *et al*., 2011). On the contrary, the strain *Tm*. sp. CB3 produced exopolysaccharide structures faster than the other cells (within only 24 h of growth, Fig. 2A). However, in the case of the *Tm*. sp. CB3 strain, the biofilm probably disseminated earlier than in the other two strains.

### Genomic comparisons

Previous studies have shown that *Thiomonas* strains originating from the Carnoulès AMD have adapted to this extremely toxic habitat by acquisition and/or loss of GEIs (Arsène‐Ploetze *et al*., 2010; Farasin *et al*., 2015; Freel *et al*., 2015). To determine whether the differences between the biofilm formation abilities and motility processes observed in this study could be due to the presence of specific GEIs present in some genomes, we investigated whether any GEIs were specific of *Tm*. sp. CB1, *Tm*. spp. CB2 or *Tm*. sp. CB3. The genomes were compared using the Regions of Genomic Plasticity (RGP) finder tool (https://www.genoscope.cns.fr/agc/microscope/compgenomics/genomicIsland.php) to identify genomic regions which have characteristics of GEIs.

First, we searched for genes involved in motility or chemotaxis in such GEIs. One GEI of around 11 kb was identified in *Tm*. spp. CB2 and CB3 but not in *Tm*. sp. CB1 (Table 1, Fig. S3). The GC content of this island is approximately 63%, which resembles that of the genome (Table 1). This GEI is located downstream of a large region (~76 kb) encoding genes involved in flagellum biosynthesis and assembly (‘flagellar locus’), which are present in all the strains tested. This GEI named ‘*che* islet’ contains the *cheW* and *cheBDR* genes, three genes encoding methyl‐accepting chemotaxis sensory transducers and a gene encoding a diguanylate cyclase/phosphodiesterase, which is probably involved in motility and biofilm regulation (Fig. S3 and Table S1). *Tm*. sp. CB1 lacking this GEI had different motility and biofilm development capacities in response to As(III) as compared to the strains *Tm*. spp. CB2 and CB3 containing the islet. Thus, this ‘*che* islet’ may be responsible for the differences of regulation in response to As(III) observed between these strains.

**Table 1:** Genomic islands characterized in this study and carrying genes potentially involved in biofilm matrix biosynthesis and/or motility (see Figs S3–S5 and Tables S1–S3 for more details)

Genomic island	Strain	Size (kB)	GC content (%)
‘*che* islet’	*Tm*. sp. CB2	11	63.1
	*Tm*. sp. CB3	11.6	63.1
‘*eps* island’	*Tm*. sp. CB1	20	63
‘*rfb* islet’	*Tm*. sp. CB2	18.9	63.7
	*Tm*. sp. CB3	22	63.81
RGP5	*Tm*. sp. CB2	29.8	57.8

One 20‐kb GEI was detected in the genome of *Tm*. sp. CB1, but not in that of *Tm*. spp. CB2 and CB3 (Table 1, Fig. S4). This GEI contains the *eps* genes forming the ‘*eps* island’, which are involved in exopolysaccharide matrix synthesis in *Herbaspirillum seropedicae* (Fig. S4 and Table S2) (Balsanelli *et al*., 2014). The GC content of this island is approximately 63%, which resembles that of the genome, and no genes involved in genome plasticity (e.g. transposases, ISs or genes involved in conjugation) were detected in the ‘*eps* island’. There was therefore no evidence that this island may have been acquired recently by *Tm*. sp. CB1.

Another GEI was present in the genome of *Tm*. spp. CB2 and CB3 but not in *Tm*. sp. CB1 (Table 1, Fig. S5). This GEI called ‘*rfb* islet’ measures 18.9 and 22 kb in the *Tm*. spp CB2 and CB3 genomes respectively. It contains the *rfbEF* and *pilT* genes and one gene encoding a glycosyl transferase (Fig. S5 and Table S3). *rfbEF* genes (also known as *rml* genes) are possibly involved in the biosynthesis of dTDP‐l‐rhamnose, a surface polysaccharide precursor (Giraud and Naismith, 2000), which is required for the attachment of *alpha*‐, *beta*‐ or *gammaproteobacteria* or biofilm formation (Rahim *et al*., 2000; Balsanelli *et al*., 2010; Michael *et al*., 2016). In *Roseobacter*, these genes are carried by a plasmid (Michael *et al*., 2016). The GC contents of the *Tm*. spp. CB2 and CB3 ‘*rfb* islets’ are 63.7% and 63.81%, respectively, which is again similar to that of the genome. However, in *Tm*. sp. CB3, this ‘*rfb* islet’ is flanked by two transposases and therefore has some of the characteristics of mobile genetic elements. Lastly, one 29.8‐kb GEI, called RGP5 (Freel *et al*., 2015), detected in *Tm*. sp. CB2 but not in the other strains tested carries a second copy of the *rfbF* gene, genes encoding glycosyltransferases, ABC transporters and type II secretion components possibly involved in O‐antigen synthesis (Freel *et al*., 2015). The GC content of this island is 57.8%, significantly lower as compared to that of the genome. These ‘*eps* island’, ‘*rfb* islet’ and RGP5 may be in part responsible for the differences in the biofilm synthesis observed in *Tm*. spp. CB1, CB2 and CB3.

### Transcriptomic analysis

The genomic comparisons highlighted some candidate genes carried on GEIs, which may confer specific characteristics to *Thiomonas* strains concerning their cell surface and may influence their biofilms architecture. To determine which of these genes could be involved in biofilm formation and regulation in response to As(III), a RNA‐seq approach was used to search for genes that are expressed during biofilm development and/or regulated in response to As(III). These experiments were performed with the *Tm*. sp. CB2 strain, as it was observed both in this study and previously (Marchal *et al*., 2011) that the biofilm polysaccharides/cells ratio increased in this strain in the presence of As(III). The biofilm cell cultures were performed in triplicates, in the same conditions than those used for crystal violet and confocal microcopy experiments: cells were incubated under stagnant conditions in the presence and in the absence of 5.33 mM As(III) during 24, 48 and 72 h. This As(III) concentration was chosen based on confocal microscopy data, as clear‐cut differences were observed at this concentration as compared to the absence of As(III) in *Tm*. sp. CB2 (Figs 1 and 2).

#### Global analysis of gene expression during biofilm development and in response to arsenite

The overall expression of 3827 genes was first studied by performing a PCA (Fig. S6). Under our experimental conditions, the three results obtained with the triplicates were similar. Although the samples obtained in the presence and absence of As(III) clustered together during the first 24 h, the patterns of expression of a large number of genes differed after longer incubation periods of 48 and 72 h, depending on whether or not As(III) was present. These observations showed that both the incubation time and the presence of As(III) affected the gene expression rates of a large number of genes. The samples obtained after 72 h in the presence of As(III) clustered together with those obtained after 48 h in the absence of As(III). The presence of As(III) therefore has an overall effect on the gene expression process, probably delaying the expression of a large number of genes. Hierarchical clustering was then performed to compare the overall patterns of gene expression occurring in the presence and absence of As(III), as well as those of each gene at the various time points. Forty groups of genes were obtained with *P*‐value < 0.001 based on the matrix counts processed using the Variance Stabilizing Transformation (VST) method (Fig. S7).

**Figure 3:**
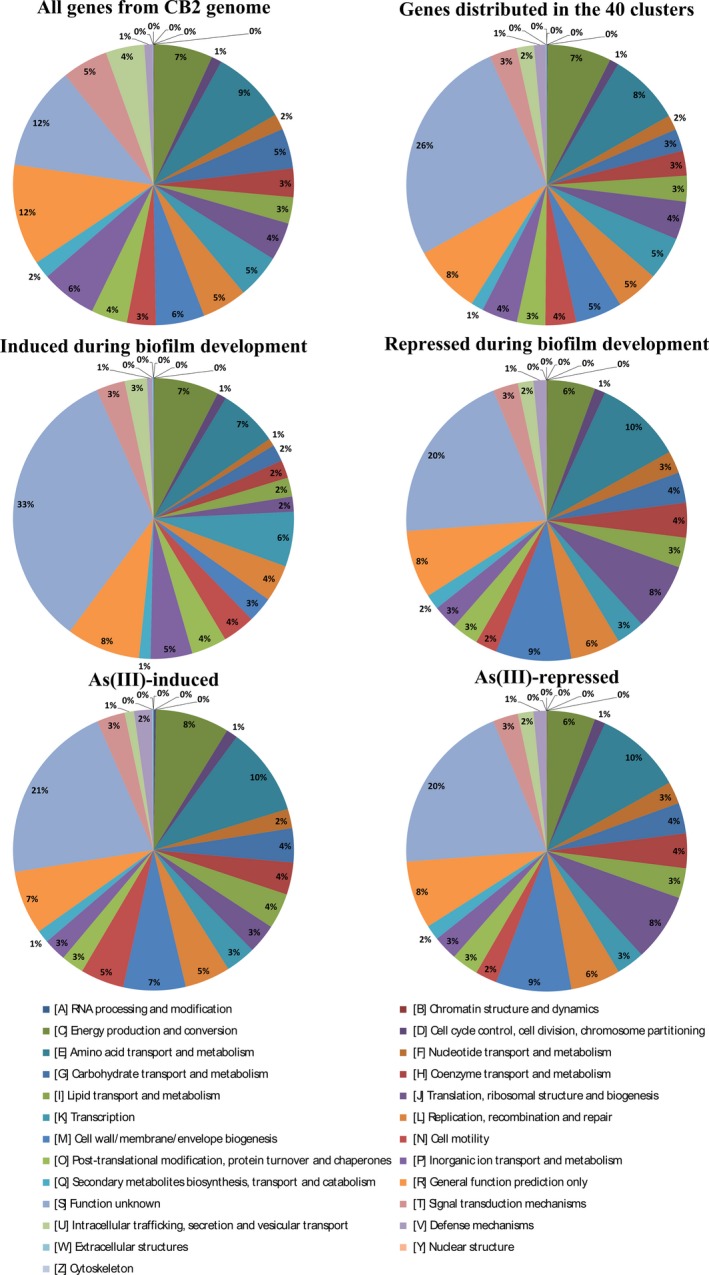
Distribution of CB2 genes found in at least one of the 40 clusters (‘regulated genes’), in ‘induced during biofilm development’, ‘repressed during biofilm development’, ‘As(III)‐induced’ or ‘As(III)‐repressed’ clusters based on the COG classification and as compared to the COG category distribution of all the genes found in the genome.

The expression of genes in eleven clusters (3, 4, 5, 7, 9, 10, 12, 13, 19, 20 and 33) decreased with time, at least in the absence of As(III) (Fig. S7). Genes with an unknown function and those involved in cell/wall membrane/envelope biogenesis, translation and amino acid metabolisms were present in larger proportions in these clusters of genes whose expression decreased during biofilm development as compared to the Cluster of Orthologous Groups (COG) category distribution of all the genes in the genome (Fig. 3). On the contrary, the levels of expression of the genes occurring in 16 clusters (1, 8, 17, 18, 21, 22, 23, 24, 26, 31, 32, 34, 35, 36, 37 and 38) increased during the biofilm development. Based on the COG classification, genes with an unknown function were present in larger proportions in these clusters of genes whose expression increased during biofilm development as compared to the COG category distribution of all the genes in the genome (Fig. 3).

**Figure 4:**
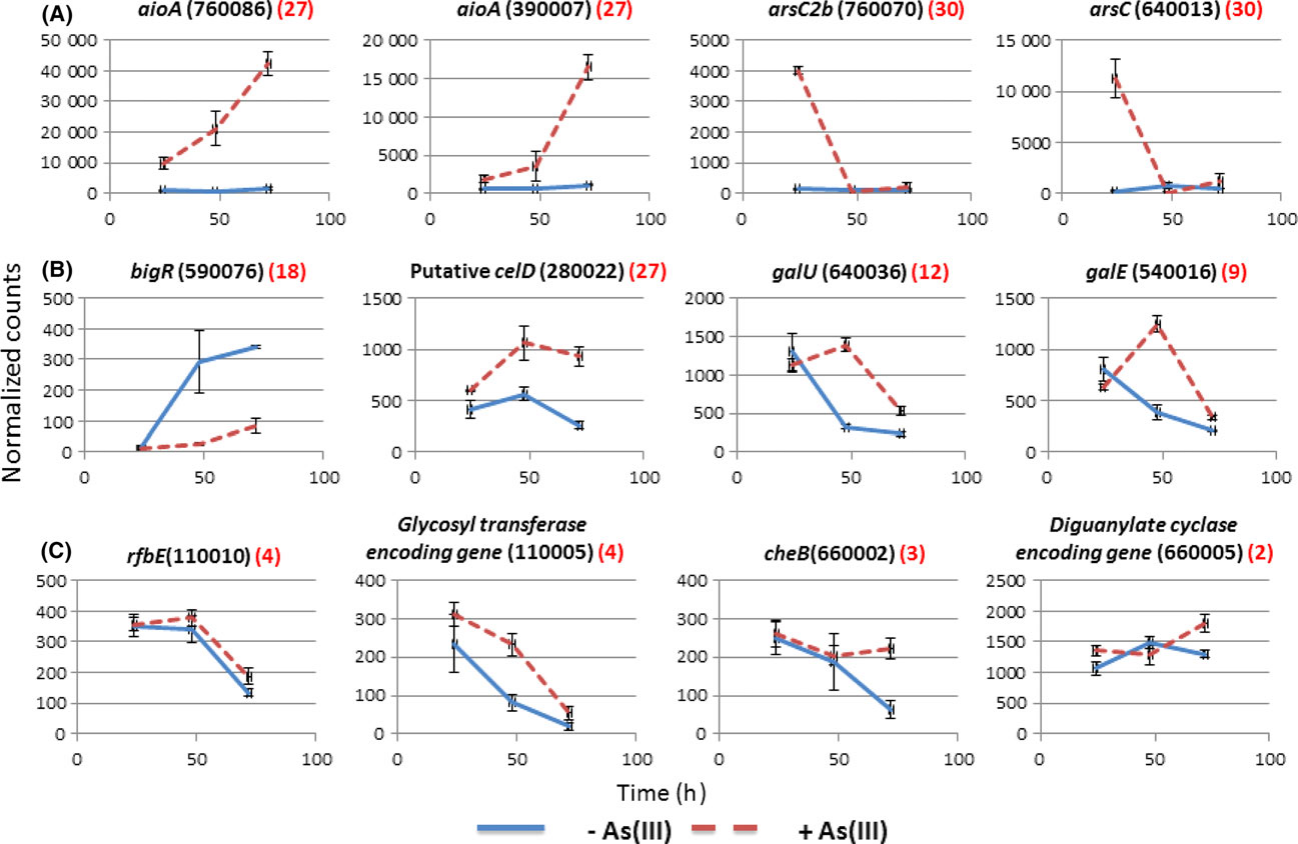
Expression patterns obtained using the RNA‐seq approach for genes involved in (A), arsenic response, (B) biofilm formation or (C) found in *che* or *rfb* islets; the black number in bracket correspond to the CDS number according to the genome sequence data (MAGE platform (http://www.genoscope.cns.fr/agc/microscope/home/)). The red number in bracket corresponds to the number of the cluster in which the gene was classified according to hierarchical classification performed in this study.

Concerning the response to As(III), 16 clusters (1, 8, 16, 17, 18, 22, 23, 24, 25, 26, 29, 32, 34, 35, 37 and 38) included 1516 genes whose patterns of expression were affected by As(III) after 24, 48 or 72 h of growth, at least at one time point during the biofilm development (Fig. S7). In comparison with the COG category distribution of all the genes in the genome (Fig. 3), genes of unknown function or involved in transcription were present in particularly large proportions in ‘arsenite‐repressed’ clusters. On the contrary, the levels of expression of the genes occurring in 17 clusters (2, 3, 4, 5, 6, 9, 11, 12, 15, 19, 20, 21, 27, 28, 30, 31 and 39) increased in the presence of As(III) (Fig. S7), at least at one time point during the biofilm development. In these clusters of genes were found, the *ars* genes (clusters 30 and 31) involved in arsenic resistance (Fig. 4A). Interestingly, cluster 27 contained genes showing higher rates of expression in the presence than in the absence of As(III) at the three time points tested during the process of biofilm development (Fig. S7). This cluster included the *aio* genes involved in As(III) oxidation (Fig. 4A) and specific phosphate transporters (Table S5), the expression of which is known to be induced in response to arsenic in several bacteria (Bryan *et al*., 2009; Cleiss‐Arnold *et al*., 2010; Andres *et al*., 2013).

#### Expression kinetics of the genes potentially involved in the biofilm regulation or architecture

Among all these regulated genes, we focused on genes potentially involved in the architecture of biofilm or in the regulation of biofilm development, as well as on genes involved in motility, a process linked to the biofilm development or dispersal processes. Several genes involved in flagellar motility, twitching motility, chemotaxis and exopolysaccharides production, were found in the clusters of genes whose expression increased during biofilm development (Table S5). However, As(III) had a negative effect on the expression of some of these genes, suggesting that these genes may play different roles in arsenite‐exposed versus non‐arsenite‐exposed biofilms (Table S5). Among these genes were found *bigR* encoding a transcriptional repressor of genes involved in biofilm development in several bacteria (Barbosa and Benedetti, 2007) and two genes encoding diguanylate cyclases, which are also known to be involved in the regulation of biofilm development (Fig. 4B). Similarly, the expression of at least 25 genes involved in cell motility (flagellar or twitching motility or chemotaxis) was induced during biofilm development in the absence of As(III), but repressed by As(III) (Table S5).

In contrast, the expression of 12 other genes involved in motility (in twitching and flagellar motility) was repressed during biofilm development, but induced in the presence of As(III) (Table S5). Similarly, the rates of expression of four genes which may be involved in EPS synthesis during biofilm development (*galE* 540016, *galU* 640036, *waaE* 30303 and a CelD‐like, 280022) and three genes uncoding diguanylate cyclase increased (at least between 24 and 48 h of growth) in the presence but not in the absence of As(III) (Table S5 and Fig. 4B). The CelD‐like gene (280022) was found in the genome in the vicinity of a diguanylate cyclase encoding gene, and both genes were included in the same arsenic‐induced cluster 27 (Table S5). These genes may have enabled the cells to produce specific components in the presence of As(III) by changing the characteristics of the biofilm matrix in the presence of the toxic metalloid. All in all, these findings show that the pattern of gene expression involved in biofilm development or dispersal differed, depending on whether As(III) was present or not.

#### Expression kinetics of genes found in the ‘rfb islet’ and ‘che islet’

Lastly, we examined specifically the patterns of expression of the genes found to occur in the three GEIs identified by genomic comparisons, which contained *rfb* genes and *che* genes (Fig. 4C and Table S5). Interestingly, the patterns of expression of the *rfb* and *che* genes varied during the biofilm development or in response to As(III). The ‘*rfb* islet’ contained 25 genes, 23 of which were detected in some of our clusters, mostly in clusters where genes are less strongly expressed in the presence than in the absence of As(III) (Table S5). However, four genes (110003, 110004, 110005 and 110010 (*rfbE*)) were included in cluster 4, and their rates of expression were slightly higher in the presence than in the absence of As(III) (Fig. 4C and Table S5). Among the 27 genes detected in RGP5 in *Tm*. sp. CB2 but not in the other strains, expression of five genes was repressed by As(III), whereas six genes showed higher rates of expression in the presence than in the absence of As(III) after 48 h of growth (Table S5). Concerning the genes found in the *che* island, we observed that the rates of expression of gene 660005, which encodes a diguanylate cyclase, *cheBDR*, 920002 (*cheW*), 920003 and 920004, which encode two methyl‐accepting chemotaxis sensory transducers, and 660006, which encodes a protein having an unknown function, were higher in the presence than in the absence of As(III) after 72 h of growth (Fig. 4C and Table S5). In contrast, loci 660001 and 920001 encoding methyl‐accepting chemotaxis sensory transducers were less strongly expressed in the presence than in the absence of As(III) (Table S5). Altogether, these results show that the expression of the genes occurring in these GEIs identified by genomic comparisons is regulated in response to As(III), which suggests that they are probably involved in the responses of *Thiomonas* sp. CB2 to As(III) during biofilm development.

## Discussion

In previous studies, we established that *Tm*. spp. 3As, CB1, CB2, CB3 and CB6 differ in their ability to degrade urea and to resist or oxidize As(III), depending on whether GEIs are present or absent (Farasin *et al*., 2015; Freel *et al*., 2015). In this study, we showed that *Tm*. spp. CB1, CB2 and CB3 present various capacities in the processes of motility and biofilm development in response to As(III). Based on crystal violet staining of the biofilm and motility tests, two groups of strains were identified. Interestingly, these two groups of strains are the same as those which emerged from previous analysis (Farasin *et al*., 2015; Freel *et al*., 2015): (i) *Tm*. sp. CB1 is mobile in the presence of As(III) and either produce relatively low amounts of biofilm and/or the biofilm is not strongly attached to the surface; (ii) *Tm*. spp. CB2 and CB3 were not mobile even in the presence of As(III) and produced larger amounts of biofilm and/or adhered more efficiently or faster to the surface than *Tm*. sp. CB1. These observations suggest that biofilm formation may compete with flagellar motility in these bacteria, and in some cases, that the motility strategy is favoured rather than biofilm formation in the presence of toxic metals. In other arsenic‐resistant bacteria, it has also been established that As(III) influences both motility and biofilm formation (Marchal *et al*., 2010; Andres *et al*., 2013). In *Herminiimonas arsenicoxydans* ULPAs1 and *Rhizobium* sp. NT26, As(III) induces swimming motility, delays the initiation of biofilm formation and reduces the amount of biofilm formed (Marchal *et al*., 2010; Andres *et al*., 2013). This pattern was therefore similar to what we observed in the present study with *Tm*. sp. CB1, but differs from what we observed here with *Tm*. spp. CB2 and CB3.

The strains *Tm*. spp. CB1 and CB2 were able to synthesize a flagellum in both the presence and absence of As(III) in the liquid growth medium (Fig. S1B). This observation suggests that the difference of motility observed in this study between these two strains was due to a functional difference between the flagella or the chemotaxis in response to As(III). In contrast, none or a very short flagellum was observed in strain CB3, whether or not As(III) was present in the liquid growth medium (Fig. S1B), suggesting a defective flagellum synthesis process in this strain.

Concerning the ability to form biofilms, we observed that the matrix composition probably differed from one strain to another. Our confocal microscopy studies showed that *Tm*. sp. CB1 produced similar amounts of exopolysaccharides to *Tm*. spp. CB2 and CB3 within 24 h. However, this strain may be more susceptible to the washing steps. It was previously established that rhizobial and burkholderial exopolysaccharides are strain‐specific heteropolymers. The great diversity of the chemical structures involved has been attributed to the expression of different enzymes in closely related bacteria (Ferreira *et al*., 2011; Janczarek, 2011). Genomic comparisons suggest that in *Thiomonas*, the ‘*eps* island’ and the ‘*rfb* islets’ may be at the origin of the differences in the matrix composition and physicochemical properties observed between *Tm*. sp. CB1 on the one hand and *Tm*. spp. CB2 and CB3 on the other hand. The exopolysaccharides produced thanks to the *eps* genes in some strains, and the *rfb* gene in other strains may be chemically different. In addition, the biofilm matrices of *Tm* sp. CB2 and CB3 are also probably different as the present confocal analysis showed differences in the architecture and in the effects of the incubation time and exposure to As(III) on biofilm formation: in the case of *Tm* sp. CB2, As(III) was found to have positive effects on the matrix formation after 72 h of growth, whereas in that of *Tm* sp. CB3, the opposite effect was observed at this time point. One GEI, RGP5, was found only in *Tm*. sp. CB2 and contains genes regulated in response to As(III) during biofilm development. Therefore, this island may be in part at the origin of the differences observed between *Tm*. spp. CB2 and CB3.

RNA‐seq experiments performed on *Tm*. sp. CB2 showed that the expression of several genes involved in biofilm development was regulated in response to As(III) in this strain. This may explain the differences observed between the polysaccharide levels in the presence and absence of As(III). The regulation of biofilm formation in response to As(III) in *Tm* sp. CB2 probably requires several regulatory cascades, as previously suggested in the case of other bacteria (Verstraeten *et al*., 2008; Janczarek, 2011; Fazli *et al*., 2014). In the present study, we observed that As(III) had negative effects on the expression of the *bigR* gene encoding a transcriptional repressor of genes involved in biofilm formation in several bacteria (Barbosa and Benedetti, 2007) and genes encoding diguanylate cyclases, known to be involved in biofilm regulation via c‐di‐GMP (Fazli *et al*., 2014; Heindl *et al*., 2014; Martínez and Vadyvaloo, 2014). As(III) may therefore regulate biofilm synthesis via these regulators in *Tm* sp. CB2. In addition, the expression of genes probably involved in the formation of the exopolysaccharides composing the matrix was induced in the presence of As(III). Among them, we highlighted *rfbE* a gene occurring on GEIs; *galE* (540016) and *galU* (640036) involved in galactose‐containing exopolysaccharides; *waaE* (30303) involved in LPS modifications, which is necessary for biofilm production in *Yersinia pestis*, and one gene encoding an Acyl‐CoA‐N‐acetyltransferase; and a CelD‐like protein (280022) involved in cellulose synthesis in *Agrobacterium tumefaciens* and attachment to surfaces (Barnhart *et al*., 2014; Liu *et al*., 2016). Our RNA‐seq experiments also showed that large proportions of genes with an unknown function were present in the clusters of genes whose expression increased or decreased during biofilm development, which suggests that other genes which have not yet been characterized may be involved in matrix formation or biofilm regulation. To test this hypothesis, it will be necessary to develop genetic tools to obtain mutants of these *Thiomonas* genes.

Biofilm formation and motility are two closely related processes, and some regulatory pathways are common to the two processes enabling bacteria to switch from a sessile lifestyle in a biofilm to the mobile planktonic lifestyle (Martínez and Vadyvaloo, 2014; Hobley *et al*., 2015; Nagar and Schwarz, 2015). In *Tm*. sp. CB2, the clusters of genes whose expression increased during biofilm development contain genes involved in flagellar motility, twitching motility and chemotaxis. Although the flagella play a role in the adhesion of some bacteria to surfaces (Stoodley *et al*., 2002; Hall‐Stoodley *et al*., 2004), the genes involved in their synthesis are normally repressed during the biofilm development (Fazli *et al*., 2014; Heindl *et al*., 2014; Martínez and Vadyvaloo, 2014). It is likely that after 24 h of growth, the transition from the planktonic to the biofilm mode had already started in *Thiomonas* sp. CB2 and that the induction of flagellar gene expression reflects the dispersion of a subpopulation. Biofilm formation, twitching and swimming motility may be co‐regulated in *Thiomonas* as in other bacteria (Guttenplan and Kearns, 2013; Tamar *et al*., 2016). We observed the induction of expression of 12 genes and repression of 25 genes found in the flagellar locus in response to As(III), which suggests that this process was tightly regulated by As(III) during the biofilm development in *Tm*. sp. CB2. It was notably observed that the *cheBDR* genes and one gene encoding a diguanylate cyclase, known to be involved in the regulation of both motility and biofilm formation and found on the ‘*che* islets’, were induced in the presence of As(III) after 72 h of growth. The induction of expression of these genes may modulate motility and perhaps increase the number of motile cells exposed to As(III), causing their dispersal as previously observed (Marchal *et al*., 2011).

In conclusion, the results obtained in this study show that biofilm biosynthesis in *Thiomonas* strains varies from one strain to another in the same species and involves several genes involved in the regulation of this process but also in motility. As biofilms and motility could be involved in niche colonization, the differences observed between the *Thiomonas* strains in these two processes may reflect different modes of adaptation to their ecological niches. On one hand, the biofilm formation may be promoted in *Tm*. spp. CB2 and CB3 in response to As(III), to protect the cells against the environmental constraints. On the other hand, *Tm*. sp. CB1 may favour the swimming process in the presence of As(III), to migrate actively to more suitable environments. *Tm*. spp. 3As, CB1 and CB6 have many GEIs in common, as for example the ‘*eps* island’, as well as functional similarities and may constitute a particular ecotype at the Carnoulès site in France (Arsène‐Ploetze *et al*., 2010; Farasin *et al*., 2015; Freel *et al*., 2015). Therefore, the three strains 3As, CB1 and CB6 may occupy different niches than strains CB2 and CB3 in the Carnoulès DMA. As biofilm formation and motility are both regulated by As(III), the present findings suggest that these processes are probably closely linked and flexible processes, which contribute importantly to the ability of these bacteria to resist to As(III).

## Material and methods

### Bacterial strains and growth conditions

*Thiomonas* spp. CB1, CB2 and CB3 were isolated from the Carnoulès AMD (Gard, France) (Bryan *et al*., 2009; Arsène‐Ploetze *et al*., 2010). They were grown *in vitro* on modified 126 medium (m126) as previously described (Bryan *et al*., 2009). As(III) was added at the concentration required from a sterile stock of 667 mM obtained with NaAsO_2_ salts (Prolabo, Fontenay‐sous‐bois, France). Biofilm cell cultures were performed in 12‐well polystyrene plates (Nunc) for crystal violet detection assays, 6‐well plates (Nunc) for RNA‐seq experiments and glass‐bottom 12‐well plates (InVitroScientific) for confocal microscopy analysis; 1 or 2.75 ml of m126 (in the case of 12‐well and 6‐well plates respectively) supplemented or not with As(III) was inoculated with exponential cell culture to a final concentration of 1%. The plates were then incubated under stagnant conditions at 30°C, and the medium was renewed after 24 h to prevent nutrient depletion.

### Crystal violet detection assay

Biofilms were grown in polystyrene 12‐well plates for 72 h as described above with three wells under each condition. The medium was removed from the wells, which were rinsed with 1 ml of physiological water (NaCl = 9 g l^−1^). The plates were then dried at 30°C for 20 min before adding 1 ml of 0.1% crystal violet and incubating them again at 30°C for 20 min. The crystal violet was removed, and the plates were rinsed again to eliminate any excess of crystal violet; 1 ml of ethanol 95% was added before re‐suspending the crystal violet diluted at 1:4 in ethanol 95% in a 96‐well plate, and the O.D was measured at 595 nm using a plate reader. Three experiments were performed with three independent cell cultures.

### Motility assays

One microliter of drop of an exponential liquid cell culture adjusted to DO ~ 0.002 was deposited on a m126 0.3% agar plate, supplemented or not with 2.67 or 5.33 mM of As(III). The plates were incubated at 30°C for 7 days.

### Transmission electron microscopy and confocal laser scanning microscopy

Transmission electron microscopy (TEM) was used to observe whether flagella were synthetized. Bacteria were deposited on a formvar grid 300 mesh, and TEM observations were made without staining as previously described (Arsène‐Ploetze *et al*., 2010). Confocal Laser Scanning Microscopy (CLSM) was used to observe the biofilm architectures. Biofilms were grown in glass‐bottom 12‐well plates as described above and stained with SYTO9 (SYTO^®^ 9 green; Life Technologies) 2.5 μM to detect bacterial cells, propidium iodide 2.5 μM to detect dead cells, and 100 μg ml^−1^ tetramethylrhodamine conjugate of ConA (Life Technologies) was used to stain glucose/mannose biofilm matrices (Life Technologies). After a 30 min period of incubation in darkness for 30 min, Z‐stacks of horizontal plane images were acquired in 1 μm steps using CLSM (Leica TCS SP8, MIMA2 microscopy platform) with a ×63 immersion lens. Two stacks were acquired randomly on three independent samples. Images (512 × 512 pixels) were recorded at an excitation wavelength of 488 nm and emission wavelengths ranging from 495 to 547 nm with SYTO9, from 657 to 757 nm with propidium iodide and at an excitation wavelength of 561 nm and emission wavelengths ranging from 567 to 589 nm with tetramethylrhodamine conjugate of ConA. Simulated 3D fluorescence projections were generated using imaris 7.0 software (Bitplane, Zürich, Switzerland). Bacterial cells (SYTO9) and mannose/glucose exopolysaccharides (ConA) were quantified from series of images using ICY (http://icy.bioimageanalysis.org/) as described previously (Sanchez‐Vizuete *et al*., 2015). Statistical analyses were performed using r (https://www.r-project.org/).

Dead cells were observed after propidium iodide labelling (Life Technologies); however, the volumes of dead cells could hardly be quantified with accuracy as the labelling with propidium iodide gave a high background noise in those samples. Attempts to detect proteins, lipids or DNA in the matrix using Ruby (FilmTracer™ SYPRO^®^; Life Technologies), FM 1‐43 (FilmTracer™ FM^®^; Life Technologies) and DDAO (7‐Hydroxy‐9H‐(1,3‐Dichloro‐9,9‐Dimethylacridin‐2‐One); LifeTechnologies) stainings, respectively, were unsuccessful. It was therefore impossible to detect the presence of any matrix compounds other than exopolysaccharides.

### Genomic comparisons

The CB2, CB1 and CB3 genomes analysed in this study were obtained previously (Freel *et al*., 2015) and integrated into the MicroScope platform (Vallenet *et al*., 2006, 2013) for analysis. The genome accession numbers of *Tm*. spp. CB2, CB1 and CB3 (EMBL database) are LK931581–LK931672, LN831666–LN831688 and LN831730–LN831775 respectively. These genomes were compared using the RGP finder tool provided with the MaGe platform (http://www.genoscope.cns.fr/agc/microscope/home/) to identify the GEIs present. The RGP Finder method starts by identifying synteny breaks between a query genome and other closely related genomes. It then searches for HGT features (tRNA hotspots, motility genes) and for the presence of any compositional bias [AlienHunter (Vernikos and Parkhill, 2006), SIGI‐HMM (Waack *et al*., 2006) and GC deviation computation] in the query genome (https://www.genoscope.cns.fr/agc/microscope/compgenomics/genomicIsland.php?act=logout).

### RNA extraction

Biofilms were grown in 6‐well plates as described above, eight plates under each condition. After a 24 h period of incubation at 30°C, the mobile phase was gently removed and the medium was renewed when further incubation was required (48 or 72 h periods of incubation). After 24, 48 or 72 h, the growth was stopped by incubating at 4°C, the mobile phase was gently removed, and 1.5 ml of physiological water was added. Biofilms were scraped on ice using a cell scraper (Biologix), centrifuged at 8000 ***g*** for 15 min at 4°C and stocked at −80°C. Three replicates from three independent cell cultures were obtained. RNA extraction was performed as described previously (Marchal *et al*., 2011) with the following modifications: cell lysis was obtained by shaking the tubes using a Retsch disruptor during three 30 s cycles and centrifuging them at 16 000 ***g*** for 5 min at 4°C. The aqueous phases were then transferred to fresh tubes and treated with Trizol as described previously (Marchal *et al*., 2011).

### RNA sequencing

Enriched mRNA was obtained from 10 μg of total RNA using the rRNA capture hybridization approach from the RiboZero kit (Epicentre, Singapore), according to the manufacturer's instructions. For high‐throughput sequencing, non‐directional cDNA libraries were prepared from enriched fragmented mRNA using the RNA sample preparation kit, set A (Illumina, San Diego, CA, USA). Fragments of cDNA of ± 150 bp, ligated with Illumina adapters and amplified per PCR, were purified from each library. Quality and quantity were confirmed on a Bioanalyzer (Agilent, Santa Clara, CA, USA). Sequencing of 51 bases was performed in single‐end mode, using an Illumina instrument (Illumina).

### Bioinformatic analysis

Reads were cleaned of adapter sequences and low‐quality sequences using an in‐house program (https://github.com/baj12/clean_ngs). Only sequences at least 25 nt in length were considered for further analysis. bowtie (v 0.12.7) using standard parameters was used to align to the *Tm*. sp. CB2 genome (Langmead *et al*., 2009). Genes were counted using HTseq‐count (parameters: ‐m intersection‐nonempty, ‐s yes, ‐t gene) (Anders *et al*., 2015).

### Statistical analysis

Count data were analysed using r version 3.1.2 (R Development Core Team, 2011) and the Bioconductor package deseq2 version 1.6.2 (Love *et al*., 2014). Data were normalized with deseq2 and the default parameter. The dispersion estimation and the statistical test for differential expression were performed with default parameters (including outlier detection and independent filtering). The generalized linear model was set with various times (24, 48 and 72 h) and two condition (arsenic versus no arsenic) as the main effect, and time × condition as the interaction. The replicate effect was also included in the model as a blocking factor. Extracted contrasts included comparisons between arsenic and no arsenic at each time point, and pairwise comparisons between time points under each condition (arsenic and no arsenic). The time × condition interactions were also tested to detect any genes with which the time effect differed from one condition to the other (arsenic or no arsenic). Raw *P*‐values were adjusted for multiple testing using the Benjamini and Hochberg (BH) procedure (Benjamini and Hochberg, 1995), and any genes with an adjusted *P*‐value lower than 0.001 were taken to be differentially expressed. A hierarchical clustering based on the correlation distance and the Ward criterion was performed to exhibit *k* = 40 groups of homogeneous genes. The homogeneity of the clusters was checked manually by inspecting the profiles of all the genes in each. Several methods were tested, and the clustering procedure was optimized to obtain homogeneous profiles within each group. The input data used for the hierarchical clustering were the VST‐transformed count matrix (Love *et al*., 2014) containing the 18 samples, and all the genes found to be differentially expressed in at least one comparison. RNA‐seq data accession number (GEO accession number): GSE85410.

## Conflict of Interest

None declared.

## Author contributions

J. F., S. K., J. D., C. P., M. E. and A. H. performed research; J. F., H. V., M.‐A. D., B. J., R. B., J.‐Y. C. and F.A.‐P. analysed data; and J.F. and F.A.‐P. wrote the manuscript.

## Supporting information


**Fig. S1.** (A) Motility test. A 1 μl droplet of an exponential culture diluted to D.O_600_ = 0.002 was deposited on soft agar (agar 0.03%) without As(III) (upper panel), or in the presence of 2.67 mM (middle panel) or 5.33 mM of As(III) (lower panel). After 7 days of growth, a motility halo was observed for *Tm*. sp. CB1 in presence of As(III), revealing that these cells are mobile in these conditions. Scale bar: 2 mm. (B) Transmission electron microscopy (TEM) photography of *Thiomonas* spp. strains cultivated in liquid medium showing the presence of flagella in CB1 and CB2 but not in CB3. The absence of motility in *Tm*. spp. CB2 observed on soft agar (Fig. S1A) as compared to CB1 was likely due to a difference in the regulation of motility. Scale bar: 2 μm.

**Fig. S2** Biofilm quantification of *Thiomonas* strains using crystal violet staining. The crystal violet staining revealed the attached cells and their biofilm matrix and was quantified by measuring the OD_595 nm_. Blue: performed without As(III); Yellow and red: performed in the presence of As(III) 2.67 and 5.33 mM, respectively. Box‐plot were drawn using r software (https://www.r-project.org/) and the package ggplot2 (https://cran.r-project.org/web/packages/ggplot2/index.html).

**Fig. S3** Synteny of the ‘*che* islet’ in *Tm*. spp. CB1, CB2 and CB3 genomes. This RGP, containing genes involved in the regulation of motility and biofilm development, is present in the *Tm*. spp. CB2 and CB3 genome (in orange) but not in the *Tm*. sp. CB1 genome. This figure was realized using the easyfig software (http://mjsull.github.io/Easyfig/; Sullivan *et al*., 2011).

**Fig. S4** Synteny of the ‘*eps* island’ involved in biofilm matrix biosynthesis of *Tm*. spp. CB1, CB2 and CB3 genomes. This RGP is found in the *Tm*. sp. CB1 genome (in blue) but not in the *Tm*. spp. CB2 and CB3 genomes. This figure was realized using the easyfig software (http://mjsull.github.io/Easyfig/; Sullivan *et al*., 2011).

**Fig. S5** Synteny of the ‘*rfb* islet’. On the contrary to the ‘*eps* island’, this RGP is found in the *Tm*. spp. CB2 and CB3 genomes (in red) but not in the *Tm*. sp. CB1 genome. This figure was realized using the easyfig software (http://mjsull.github.io/Easyfig/; Sullivan *et al*., 2011).

**Fig. S6** First two components of a Principal Component Analysis, with percentages of variance associated with each axis. The first principal component (PC1) separated samples from the different biological conditions, meaning that the biological variability is the main source of variance in the data. Blue and Red: replicates obtained after growth in the absence or in the presence of arsenite 5.33 mM, respectively.

**Fig. S7** Patterns of gene expression changes during As(III) exposure and during biofilm development. Expression graphs of the 40 clusters obtained by plotting the normalized counts (*y*‐axis) at each of the three time points (*x*‐axis). The average for all genes in each cluster is highlighted by a blue line (in absence of As(III)) or a red line (in the presence of 5.3 mM As(III)).

**Table S1.** Details of the region containing the ‘*che* islet’ in *Tm*. spp. CB2 and CB3.

**Table S2.** Details of the region containing the ‘*eps* island’ in *Tm*. sp. CB1.

**Table S3.** Details of the region containing the ‘*rfb* islet’ found in the *Tm*. spp. CB2 and CB3.

**Table S4.** Number of up‐, down‐ and total number of differentially expressed features for each comparison.

**Table S5.** Effect of arsenic on expression of 138 genes involved in chemotaxis, motility, biofilm development and its regulation.
