# Supplementary figures and images for: Comparison of biofilm formation and motility processes in arsenic‐resistant Thiomonas spp. strains revealed divergent response to arsenite
Source: Microb Biotechnol. 2017 Feb 7;10(4):789–803. doi: 10.1111/1751-7915.12556 (PMC5481541; doi:10.1111/1751-7915.12556)

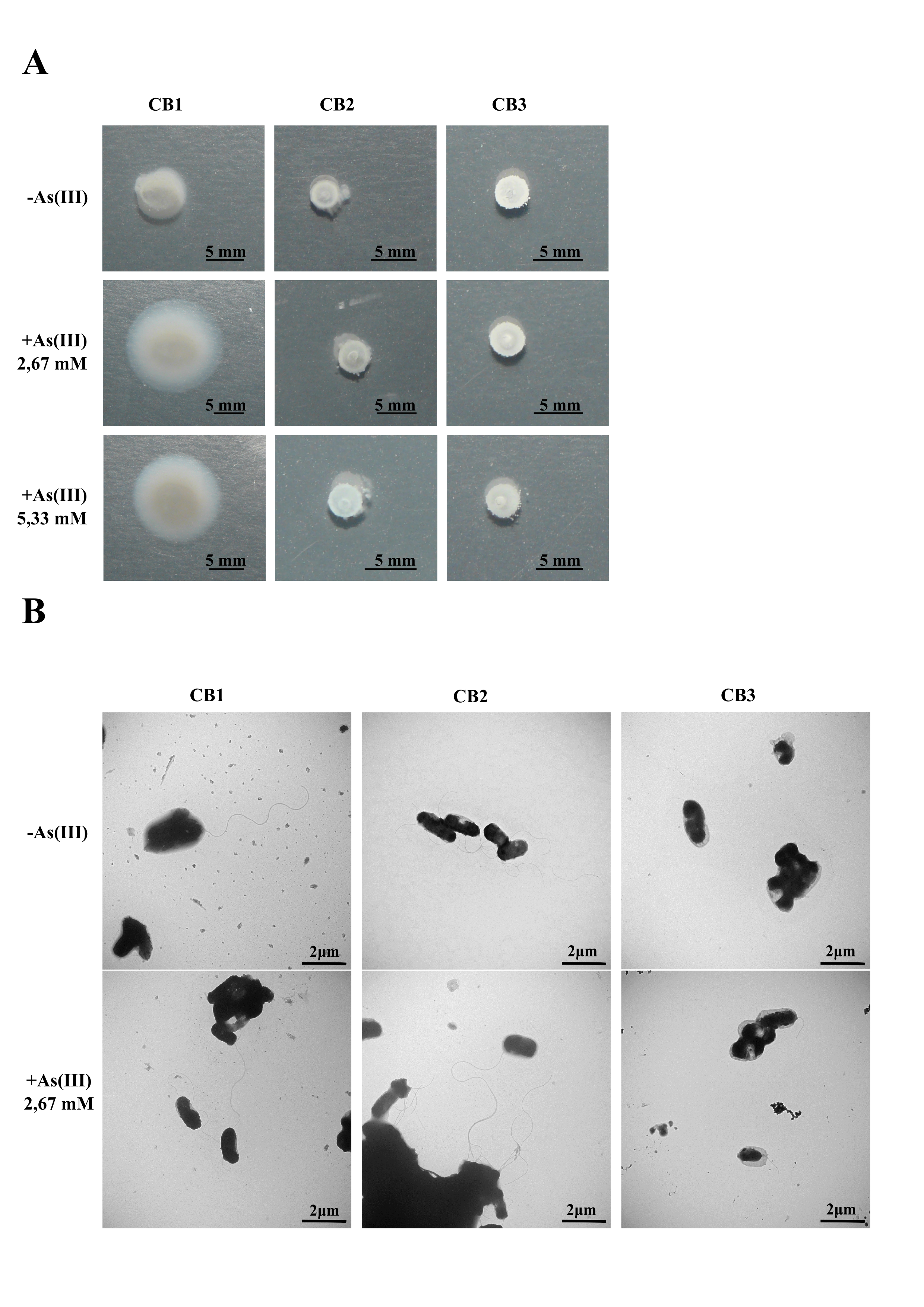

Supplement: Supplementary file 1 — Fig. S1. (A) Motility test. A 1 μl droplet of an exponential culture diluted to D.O600 = 0.002 was deposited on soft agar (agar 0.03%) without As(III) (upper panel), or in the presence of 2.67 mM (middle panel) or 5.33 mM of As(III) (lower panel). After 7 days of growth, a motility halo was observed for Tm. sp. CB1 in presence of As(III), revealing that these cells are mobile in these conditions. Scale bar: 2 mm. (B) Transmission electron microscopy (TEM) photography of Thiomonas spp. strains cultivated in liquid medium showing the presence of flagella in CB1 and CB2 but not in CB3. The absence of motility in Tm. spp. CB2 observed on soft agar (Fig. S1A) as compared to CB1 was likely due to a difference in the regulation of motility. Scale bar: 2 μm. [file MBT2-10-789-s001.tif]

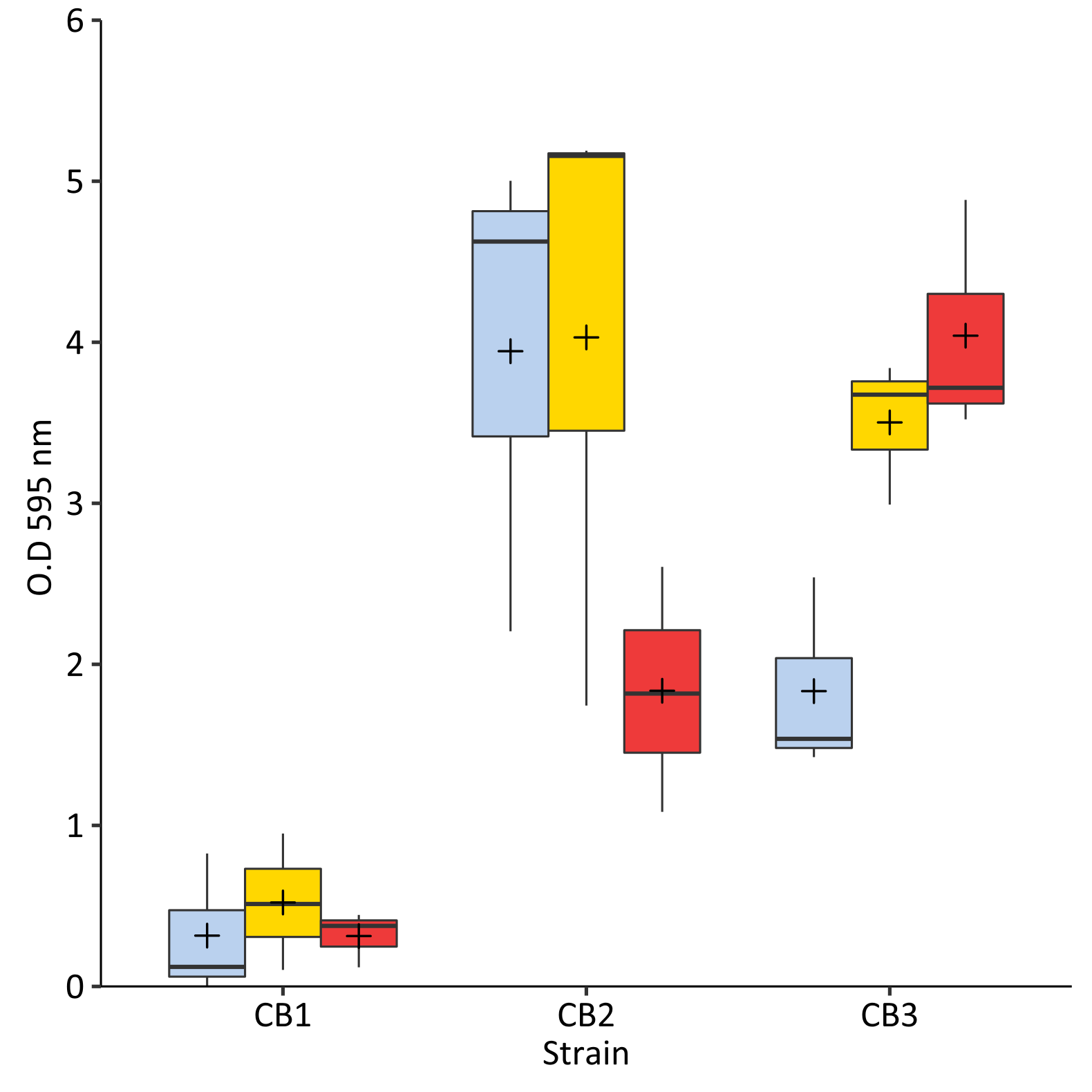

Supplement: Supplementary file 2 — Fig. S2 Biofilm quantification of Thiomonas strains using crystal violet staining. The crystal violet staining revealed the attached cells and their biofilm matrix and was quantified by measuring the OD 595 nm. Blue: performed without As(III); Yellow and red: performed in the presence of As(III) 2.67 and 5.33 mM, respectively. Box‐plot were drawn using r software (https://www.r-project.org/) and the package ggplot2 (https://cran.r-project.org/web/packages/ggplot2/index.html). [file MBT2-10-789-s002.tif]

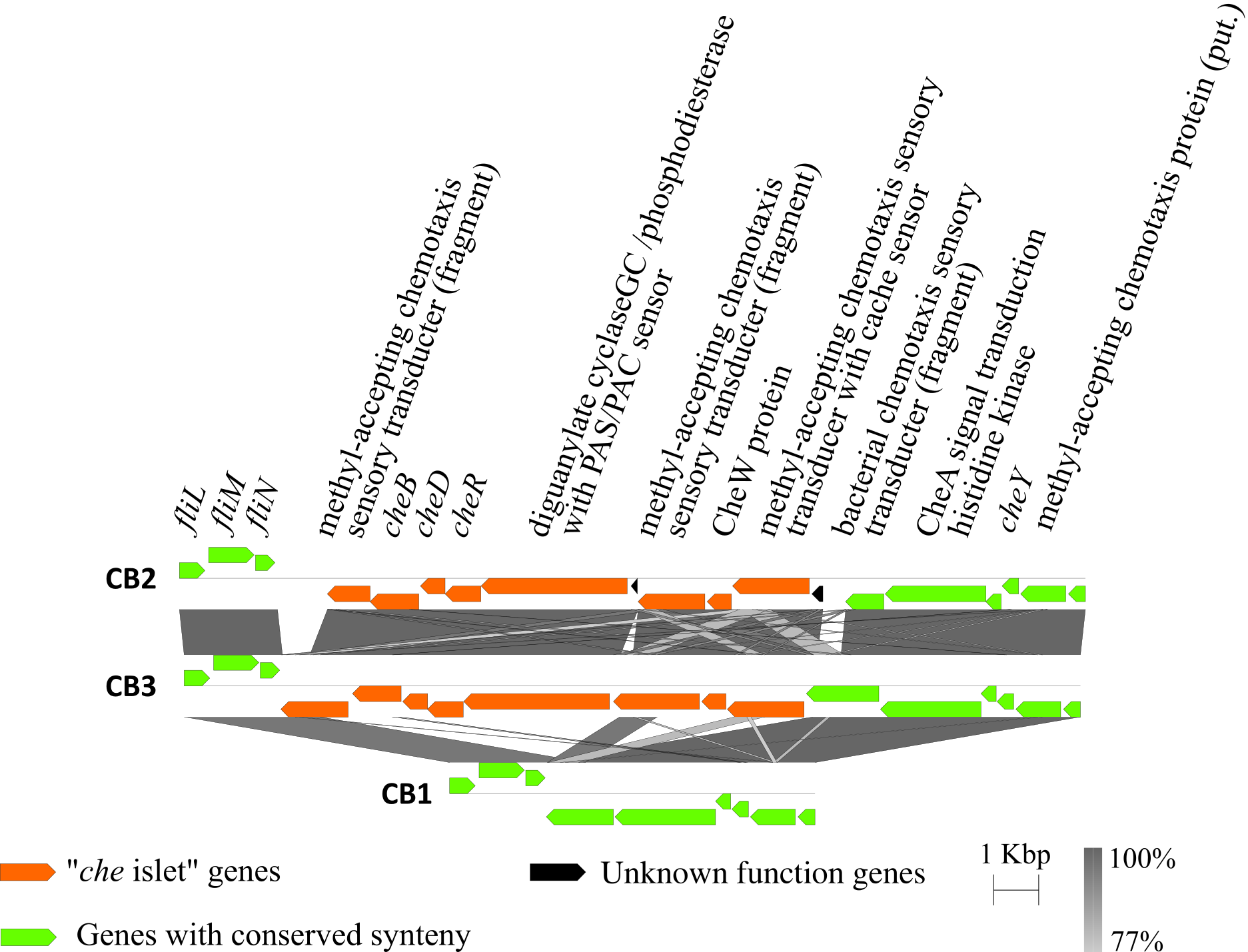

Supplement: Supplementary file 3 — Fig. S3 Synteny of the ‘che islet’ in Tm. spp. CB1, CB2 and CB3 genomes. This RGP, containing genes involved in the regulation of motility and biofilm development, is present in the Tm. spp. CB2 and CB3 genome (in orange) but not in the Tm. sp. CB1 genome. This figure was realized using the easyfig software (http://mjsull.github.io/Easyfig/; Sullivan et al., 2011). [file MBT2-10-789-s003.tif]

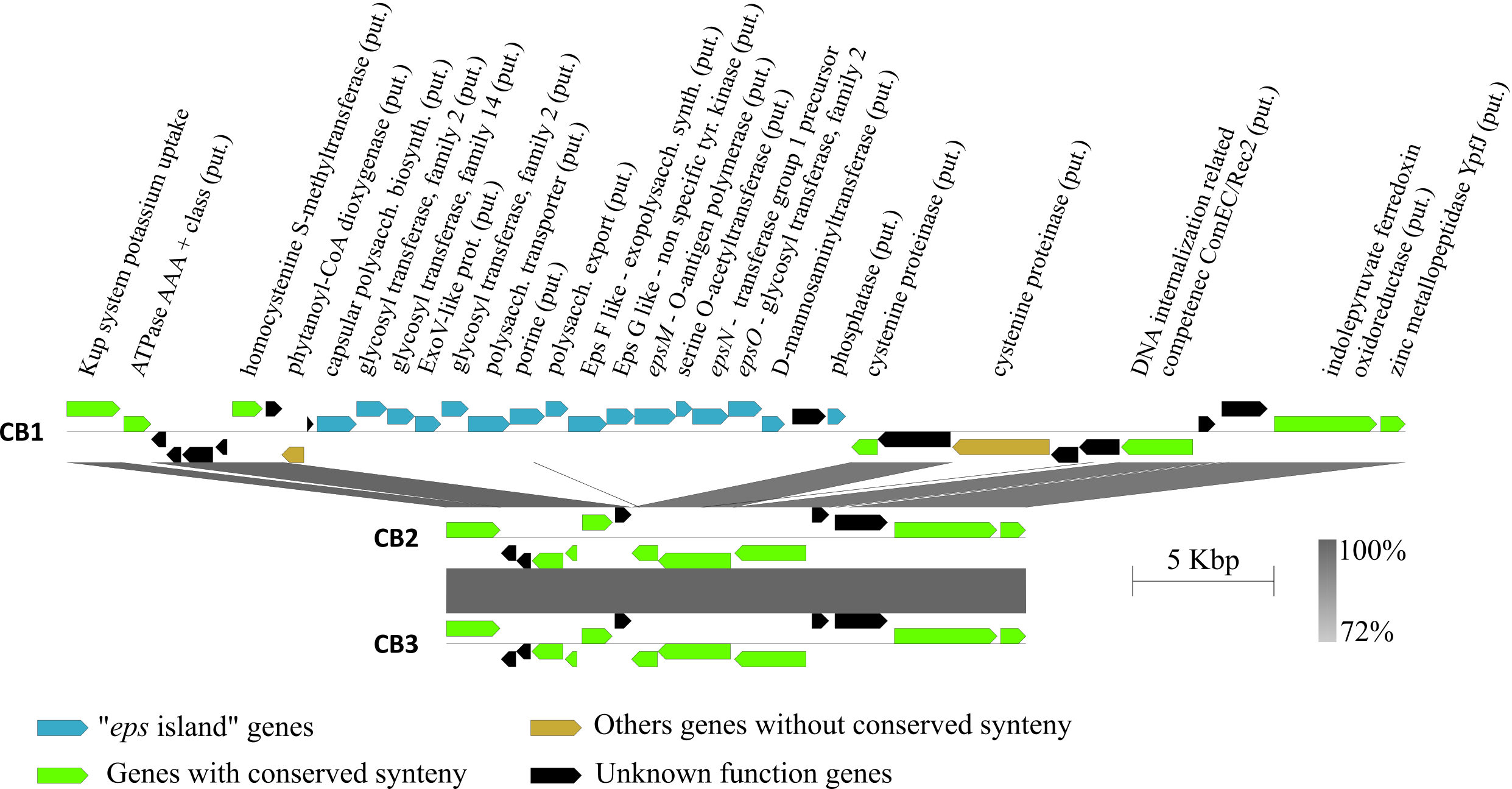

Supplement: Supplementary file 4 — Fig. S4 Synteny of the ‘eps island’ involved in biofilm matrix biosynthesis of Tm. spp. CB1, CB2 and CB3 genomes. This RGP is found in the Tm. sp. CB1 genome (in blue) but not in the Tm. spp. CB2 and CB3 genomes. This figure was realized using the easyfig software (http://mjsull.github.io/Easyfig/; Sullivan et al., 2011). [file MBT2-10-789-s004.tif]

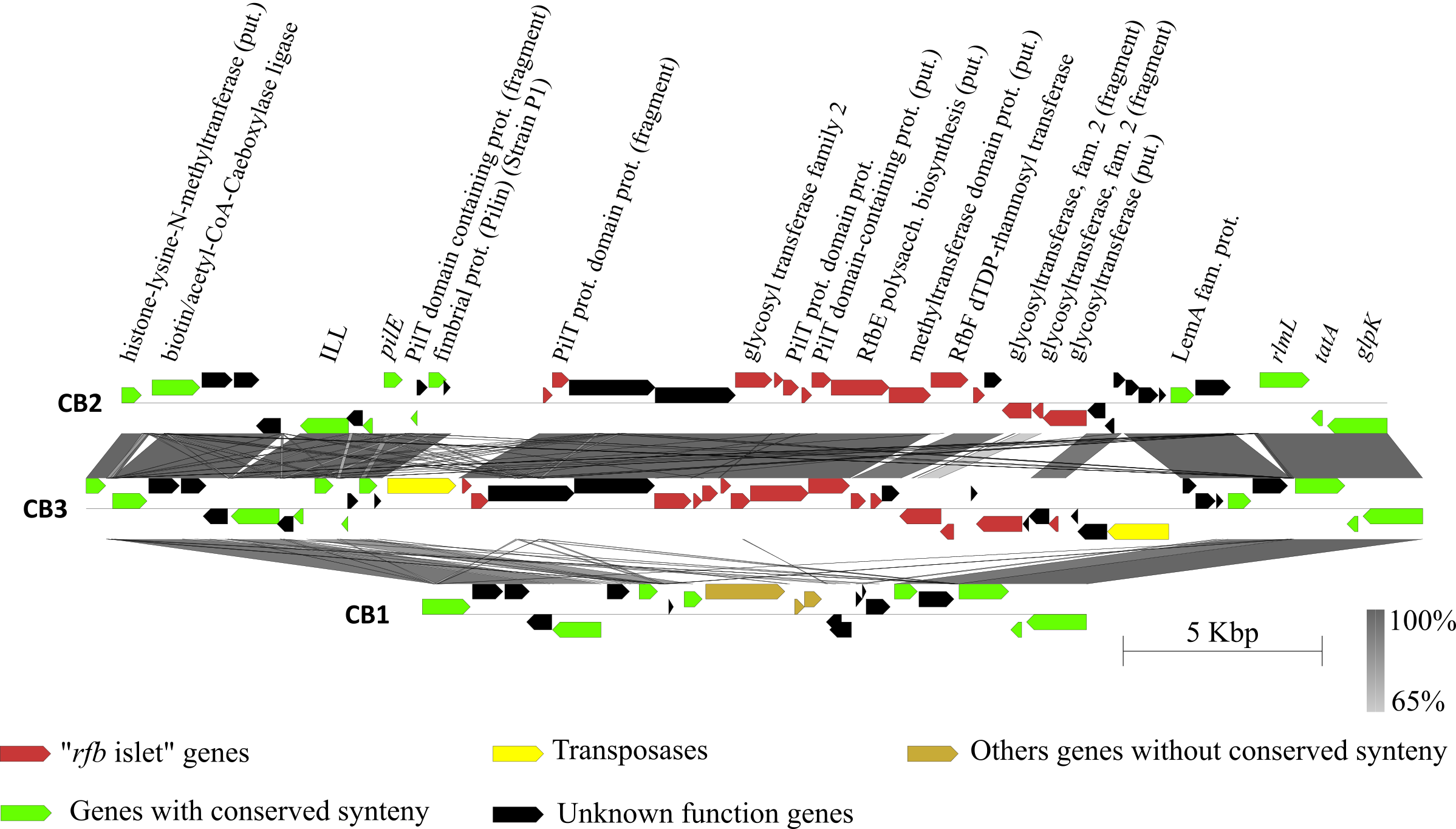

Supplement: Supplementary file 5 — Fig. S5 Synteny of the ‘rfb islet’. On the contrary to the ‘eps island’, this RGP is found in the Tm. spp. CB2 and CB3 genomes (in red) but not in the Tm. sp. CB1 genome. This figure was realized using the easyfig software (http://mjsull.github.io/Easyfig/; Sullivan et al., 2011). [file MBT2-10-789-s005.tif]

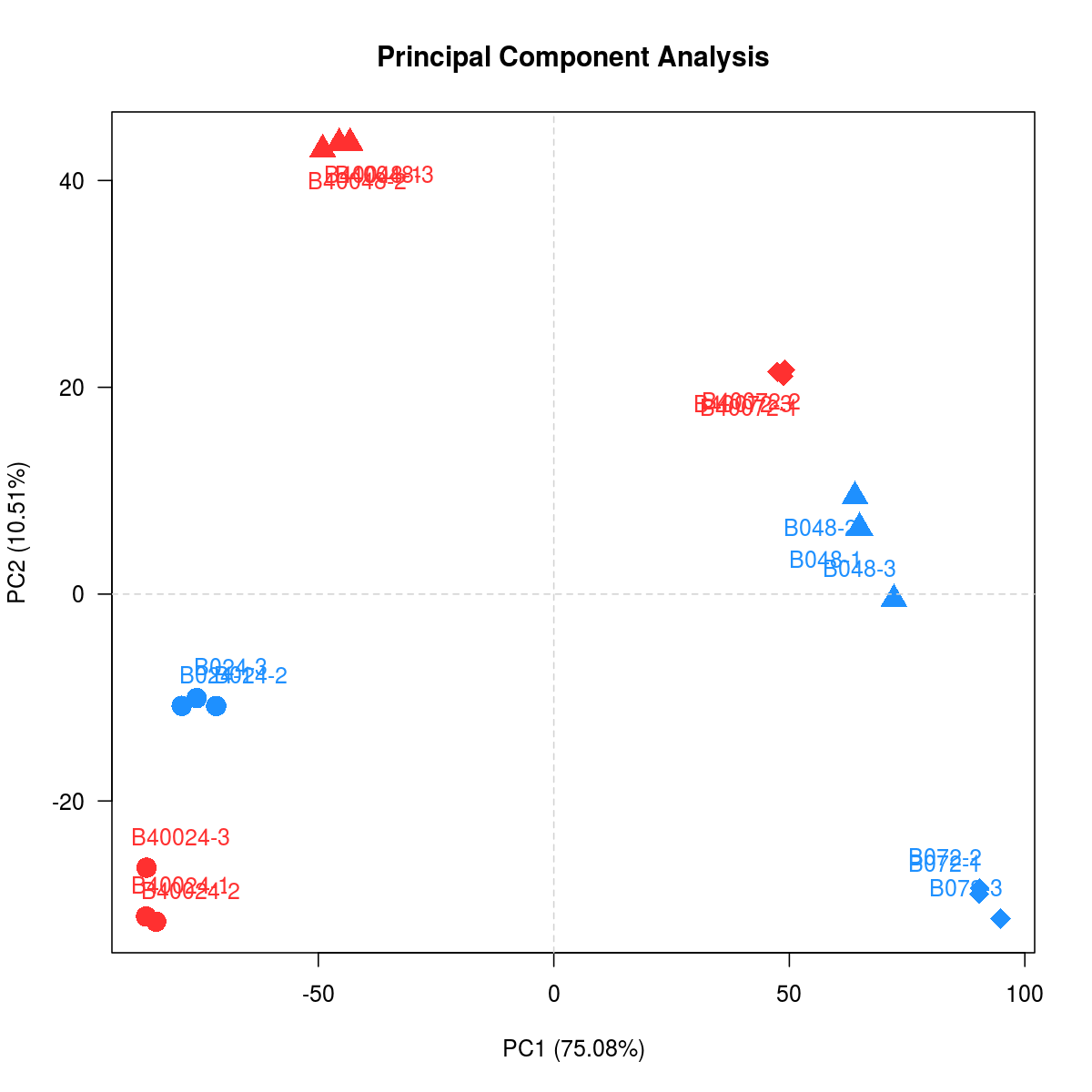

Supplement: Supplementary file 6 — Fig. S6 First two components of a Principal Component Analysis, with percentages of variance associated with each axis. The first principal component (PC1) separated samples from the different biological conditions, meaning that the biological variability is the main source of variance in the data. Blue and Red: replicates obtained after growth in the absence or in the presence of arsenite 5.33 mM, respectively. [file MBT2-10-789-s006.tiff]

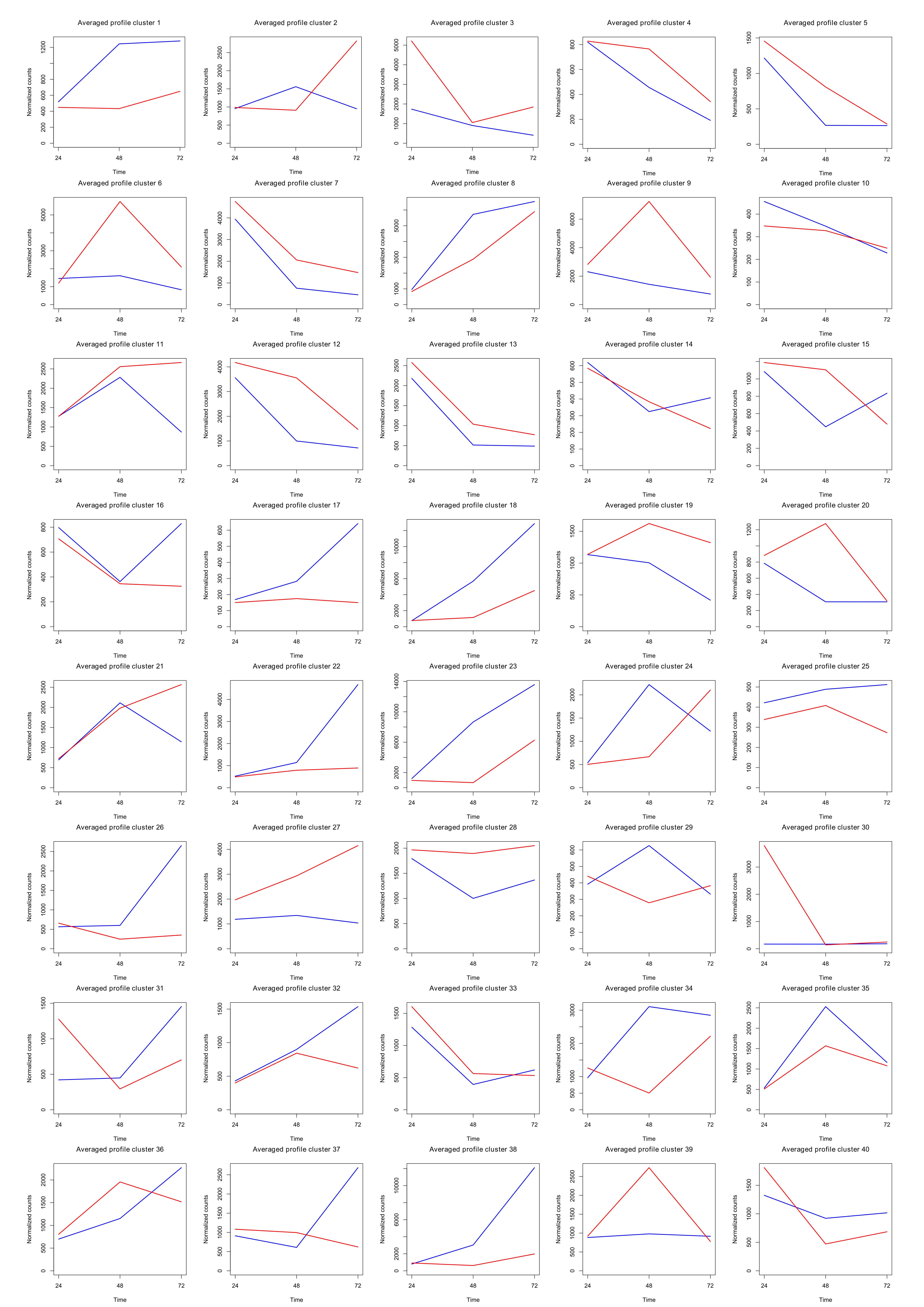

Supplement: Supplementary file 7 — Fig. S7 Patterns of gene expression changes during As(III) exposure and during biofilm development. Expression graphs of the 40 clusters obtained by plotting the normalized counts (y‐axis) at each of the three time points (x‐axis). The average for all genes in each cluster is highlighted by a blue line (in absence of As(III)) or a red line (in the presence of 5.3 mM As(III)). [file MBT2-10-789-s007.tif]
