# Supplementary material for: Comparison of biofilm formation and motility processes in arsenic‐resistant Thiomonas spp. strains revealed divergent response to arsenite
Source: Microb Biotechnol. 2017 Feb 7;10(4):789–803. doi: 10.1111/1751-7915.12556 (PMC5481541; doi:10.1111/1751-7915.12556)
Supplement: Supplementary file 11 — Table S4. Number of up‐, down‐ and total number of differentially expressed features for each comparison. [file MBT2-10-789-s011.docx]

**Supplementary table 4:** Number of up-, down- and total number of differentially expressed features for each comparison.

| Test vs Ref | # down | # up | # total |
| --- | --- | --- | --- |
| a400-24h vs a0-24h | 252 | 372 | 624 |
| a400-48h vs a0-48h | 1435 | 1494 | 2929 |
| a400-72h vs a0-72h | 1268 | 1196 | 2464 |
| a0-48h vs a0-24h | 1522 | 1476 | 2998 |
| a0-72h vs a0-24h | 1547 | 1587 | 3134 |
| a0-72h vs a0-48h | 1109 | 1238 | 2347 |
| a400-48h vs a400-24h | 1275 | 1124 | 2399 |
| a400-72h vs a400-24h | 1413 | 1374 | 2787 |
| a400-72h vs a400-48h | 1271 | 1269 | 2540 |
| (a400-72h vs a0-72h) vs (a400-24h vs a0-24h) | 1238 | 1116 | 2354 |
| (a400-72h vs a0-72h) vs (a400-48h vs a40-48h) | 1185 | 1203 | 2388 |
| (a400-48h vs a0-48h) vs (a400-24h vs a0-24h) | 962 | 1000 | 1962 |
